# From Source to Utilization: A Review of Influencing Factors and Safety Assessment for High-Value Utilization of Sweet Potato (*Ipomoea batatas* L.) Leaves

**DOI:** 10.3390/foods15142563

**Published:** 2026-07-21

**Authors:** Menghuan Sun, Xiaojing Jiang, Yulu Ma, Liang Wang, Ming Zhu, Jun Xing, Jingyang Hong

**Affiliations:** 1 College of Smart Agriculture, Xinjiang University, Urumqi 830046, China; 107552403518@stu.xju.edu.cn (M.S.); jiangxj0303@163.com (X.J.); 13150252123@163.com (Y.M.); wl1390593786@163.com (L.W.); minna0130@163.com (M.Z.); xjdxzl01@163.com (J.X.); 2 Silk Research Institute (Fruit and Vegetable Processing), Urumqi 830046, China

**Keywords:** sweet potato leaves, bioactive components, safety, high-value utilization

## Abstract

Sweet potato leaves (SPLs), with an annual production of millions of tons, are rich in polyphenols and other bioactive components, yet remain largely discarded or underutilized. This review proposes, for the first time, a “source–process–safety” full-chain cascade framework that elucidates three key aspects: how upstream factors (genotype and harvest period) predetermine raw material quality baselines; how midstream processing mediates the balance between activity retention and flavor improvement; and how downstream applications are subject to the decisive thresholds of heavy metal safety and regulatory compliance. Collectively, this framework provides a theoretical foundation and practical guidance for the high-value utilization of sweet potato leaves.

## 1. Introduction

Sweet potato (*Ipomoea batatas* L.), a member of the Convolvulaceae family, is the sixth most important food crop worldwide. It is cultivated in over 50 countries, primarily in Asia and Africa [1]. According to FAO data (2024), global sweet potato production reached 112.42 million metric tons, with China contributing 45.9% (51.57 million tons) [2]. Based on the estimated ratio of 0.29–0.42 tons of fresh leaves per ton of tubers, China alone generates approximately 15 to 21.7 million tons of sweet potato leaf (SPL) by-products annually [3,4].

Despite the abundant biomass, SPLs remain largely underutilized. Due to their flavor characteristics and the lack of systematic processing frameworks, they are either discarded or used only as low-value animal feed [5]. This underutilization stands in stark contrast to the exceptional nutrient density of SPLs: their protein content reaches up to 4.8 g/100 g fresh weight (FW), nearly twice that of spinach (2.6 g/100 g FW); their calcium concentration (up to 174 mg/100 g FW) is approximately double that of spinach (99 mg/100 g FW) [6,7,8]. Moreover, data from the Chinese Center for Disease Control and Prevention indicate that β-carotene levels in SPL are comparable to, or even exceed, those found in carrots [9,10]. In recognition of this robust nutritional profile, the Chinese Nutrition Society has explicitly included sweet potato leaves as a recommended dark green vegetable in the Dietary Guidelines for Chinese Residents [11]. Beyond basic nutrition, SPLs synthesize a diverse array of bioactive compounds, predominantly polyphenols, flavonoids, and polysaccharides; extensive in vitro and in vivo studies have consistently demonstrated that these components confer significant antioxidant, anti-inflammatory, hypoglycemic, and hypolipidemic activities [12,13].

Given the outstanding nutritional and bioactive profile of sweet potato leaves (SPLs), they have attracted growing research interest as a potential functional food ingredient. However, current investigations remain largely confined to laboratory-scale ideal conditions and have yet to address critical translational hurdles, including novel food ingredient applications, Generally Recognized as Safe (GRAS) certification, and industrial-scale processing feasibility. Consequently, the existing literature is predominantly composed of fragmented case studies that, while informative, lack a systematic whole-chain perspective linking upstream agronomic variables, midstream processing parameters, and downstream safety and regulatory requirements.

To bridge this gap, this review constructs an integrated framework of “source control—processing preparation—potential application—safety compliance”. In contrast to traditional reviews that treat agronomic cultivation, processing technologies, and bioactive functions as separate domains, our framework first establishes upstream genotype, harvest stage, and cultivation conditions as the core determinants of raw material quality. Second, it systematically elucidates the balance mechanism between flavor release and bioactive compound retention during processing. Finally, based on the quality variations arising from the aforementioned multiple factors, this framework moves beyond the parallel classification of food, feed, and industrial raw materials, and instead proposes a hierarchical priority strategy for high-value SPL utilization according to the thermal sensitivity thresholds and bioconversion efficiency of different components, thereby providing both a theoretical foundation and actionable pathways for the high-value transformation of this agricultural by-product. On the basis of this chain-based logic, this review is the first to incorporate national regulatory frameworks and allergenicity assessment into the evaluation system, and to reassess the industrial prospects of SPL from the perspective of commercial feasibility.

## 2. Characteristics and Influencing Factors of SPL

**Figure 1:**
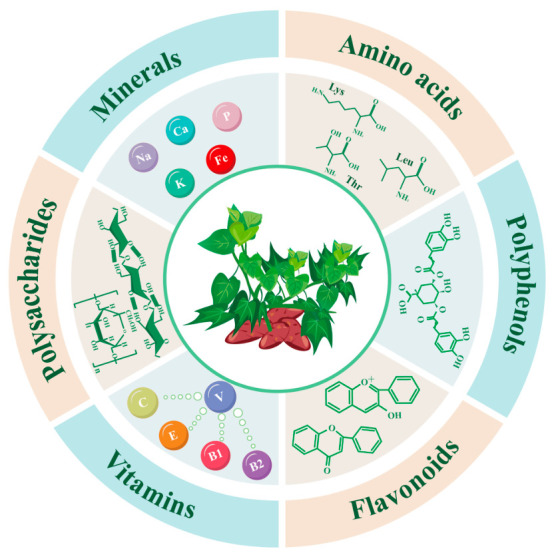
Overview of the primary nutritional and bioactive components in sweet potato leaves.

Figure 1 summarizes the complex nutrient and bioactive compound composition of SPL. This section systematically examines these intrinsic properties and their modulating factors, with particular emphasis on the effects of cultivar, harvest stage, and cultivation conditions on quality variation.

### 2.1. Composition Basic Nutrition and Bioactive Compounds of SPL

#### 2.1.1. Crude Protein

Research indicates that the crude protein content of SPL ranges from 2.61 ± 0.58 to 4.85 ± 0.02 g/100 g FW, a level comparable to that of spinach and kale [14]. Amino acid profiling across 17 cultivars confirms the presence of all essential (EAAs) and non-essential amino acids (NEAAs), consistent with the findings of Ishida [14,15]. Specifically, leucine is the predominant EAA, while glutamic acid is the most abundant NEAA, the latter of which imparts a characteristic mushroom-like umami flavor to the leaves [14]. However, the literature regarding the limiting amino acids (LAAs) of SPL proteins shows significant discrepancies: studies employing the FAO/WHO/UNU (1985) standard identified lysine as the first LAA (AAS 76–84%), whereas recent evaluations based on the WHO/FAO (2007) criteria pinpoint sulfur-containing amino acids (methionine + cysteine) as the primary limiting factor (AAS 32.58%) [5,15]. These variations are likely attributable to evolving evaluation standards and genotypic differences among cultivars. Despite the limitations in amino acid scores, the nutritional quality of SPL protein can be optimized through dietary complementation with staples such as cereals, which are rich in sulfur-containing amino acids but deficient in lysine [16].

#### 2.1.2. Dietary Fiber

Research consistently shows that fiber content in SPL is uniform across cultivars, with stems exhibiting higher levels than leaves [14,15]. This disparity is attributed to the higher lignin content in the stems. Structurally, leaves possess a greater proportion of soluble dietary fiber (SDF), which acts as a prebiotic to be fermented by beneficial gut microbiota, thereby modulating intestinal microecology, enhancing gut health, and reducing blood glucose. Conversely, the stems are richer in insoluble dietary fiber (IDF), which promotes intestinal peristalsis and effectively alleviates constipation [17]. However, SDF accounts for only approximately 10% of the total fiber in SPL [15]. This high proportion of IDF imparts a coarse, fibrous texture, resulting in poor palatability when consumed fresh. In food processing excessive SPL supplementation disrupts the gluten network, leading to reduced specific volume, increased hardness, and surface cracking. Studies indicate that while addition levels of 2–10% are acceptable, concentrations exceeding 16% cause significant quality deterioration [16]. These textural and structural challenges remain a primary constraint on the large-scale utilization of SPL in staple food products.

#### 2.1.3. Micronutrient

In addition to the trace elements listed in Table 1, SPLs contain vitamin E, vitamin B2, and minerals including Na, Se, Mg and Zn [14,15,18,19]. While the vitamin and mineral profiles are generally consistent across various cultivars, purple-leaf varieties exhibit significantly lower Na levels compared to green-leaf ones, despite having comparable K content [14]. Given that an elevated K/Na ratio is associated with a reduced risk of atherosclerosis and hypertension, high-K/low-Na cultivars such as “Guangcaishu” are nutritionally preferable [14,20]. However, high micronutrient content does not necessarily translate into high intestinal absorption. The bioavailability of minerals—particularly Na Zn, and Mg—is markedly hindered by endogenous anti-nutritional factors [21,22]. For instance, the average oxalate content in sweet potato leaves is 530.20 mg/100 g fresh weight (FW); although lower than that of spinach (970.00 mg/100 g FW), it is 26 times higher than that of kale [22,23]. These oxalates bind with calcium to form insoluble calcium oxalate, thereby reducing Ca absorption [24]. Consequently, similar to spinach, pre-consumption blanching of sweet potato leaves can effectively enhance mineral bioavailability, a finding corroborated by the research of Mwanri [22]. All the aforementioned nutrients are systematically summarized in Table 1.

#### 2.1.4. Major Bioactives: Polyphenols

In terms of functional components, polyphenols constitute the most characteristic bioactive constituents of SPL, primarily comprising phenolic acids (predominantly caffeoylquinic acid derivatives) and flavonoids (including flavonols, flavones, flavanones, isoflavones, anthocyanins, and tannins) [12,30,31,32,33]. Among these, 3,5-dicaffeoylquinic acid (3,5-diCQA) is the most abundant monomeric compound and exhibits the strongest antioxidant activity, being recognized as the signature antioxidant in SPL [34]. However, reported concentrations of identical compounds exhibit substantial disparities across the literature—total phenolic content ranges from 2.90–375.44 mg GAE/g, total flavonoid content from 0.6 to 127.12 mg RE/g, and 3,5-dicaffeoylquinic acid spans four orders of magnitude (Table 2 and Table 3). These dramatic variations are not attributable solely to analytical artifacts, but rather reflect the systematic influence of upstream factors—including genotype, harvest period, and environmental conditions—on raw material quality (detailed in Section 2.2), while simultaneously exposing the more fundamental issue of lacking standardized detection frameworks in current research.

### 2.2. Key Factors Affecting the Content and Quality of SPL

The marked compositional variations presented in Section 2.1—ranging from over an order-of-magnitude differences in total phenolic content across cultivars to several orders-of-magnitude fluctuations in signature compounds such as 3,5-dicaffeoylquinic acid—are not random, but are systematically driven by three categories of upstream factors: genotype, plant organ and harvest timing, and environmental conditions [39,40,41]. Elucidating these determinants is essential not only for understanding the fundamental biology of sweet potato leaves, but also for establishing rational raw material selection criteria for downstream processing.

#### 2.2.1. Genotype (Cultivar Variation)

Regarding functional components, plant genotype dictates the composition and abundance of specific secondary metabolites. A systematic comparison of five purple-fleshed sweet potato genotypes revealed distinct phytochemical profiles under identical cultivation conditions. Specifically, genotype ‘2019-11-2’ exhibited the highest concentration of anthocyanins in leaves during the vegetative stage; genotype ‘purple-purple’ accumulated peak levels of caffeoylquinic acid derivatives during the tuberous root initiation stage; whereas the U.S. genotype ‘08-21P’ showed superiority in carotenoid accumulation [42]. At the molecular level, proteomic and transcriptomic analyses provide direct evidence for these genotypic variations. Comparative proteomics between sweet potato leaves and storage roots identified 4321 non-redundant proteins, comprising 3143 leaf-specific and 2928 root-specific proteins. The leaf proteome is primarily enriched in pathways associated with photosynthesis and protein synthesis. Concurrently, related genes are significantly enriched in pathways involving sugar metabolism, amino acid biosynthesis, and secondary metabolism, thereby elucidating the molecular basis by which genetic variation drives nutritional discrepancies at both transcriptional and translational levels [43]. Consequently, targeted breeding for cultivars rich in functional components is highly feasible from a genetic standpoint. Indeed, grey relational analysis has already identified cultivars with superior comprehensive nutritional profiles, such as ‘Tainong 71’, ‘Fu22’, and ‘Ningcai’ [5].

#### 2.2.2. Plant Organs and Harvesting Periods

The accumulation of nutritional components in sweet potatoes is significantly influenced by both plant organs (leaves, stems, and stalks) and harvesting periods. Regarding organ specificity, leaves generally exhibit higher concentrations of proteins, vitamins, and carotenoids than stems and stalks. It has been reported that the contents of β-carotene, along with vitamins B2, C, and E, in leaves surpass those in other parts [15]. Similar disparities in nutritional compounds have been documented between stem tips and lower stem sections [14]. Fundamentally, these inter-organ variations stem from differences in cell wall composition and metabolic specialization: leaves are primary sites for photosynthesis and protein synthesis, whereas stems and petioles serve support and transport functions, characterized by higher fiber content and a greater degree of lignification [44].

Harvesting timing constitutes another critical determinant of nutritional quality. Research involving three distinct harvest stages—vegetative stage (VS, 8 WAP), tuberous root initiation stage (TIS, 12 WAP), and tuberous root maturation stage (TMS, 16 WAP)—demonstrates that different genotypes reach their nutritional peaks at different times. For instance, genotype ‘2019-11-2’ attained its maximum anthocyanin concentration at 8 WAP, while ‘purple-purple’ reached its peak phenolic acid content at 12 WAP, indicating a significant interaction between genotype and harvest period [27]. Furthermore, evidence suggests that photoperiod (long-day vs. short-day) affects the accumulation of hydroxycinnamic and hydroxybenzoic acids, illustrating that the dynamics of secondary metabolites are co-regulated by developmental stages and environmental signals [29]. Consequently, harvesting schedules in practical production should be optimized based on target nutritional components to achieve precision harvesting.

#### 2.2.3. Environmental Conditions

Overall, the impact of environmental conditions (e.g., climate, soil, cultivation methods, and water stress) on the nutritional profile of sweet potato stems and leaves is generally secondary to genotypic effects; however, these factors can exert significant regulatory effects under specific circumstances. Light exposure is a critical environmental factor influencing the accumulation of phenolic acids and flavonoids. Under long-day conditions, the contents of p-coumaric and sinapic acids in sweet potato leaves are significantly higher than those under short-day treatments [29]. Additionally, hydroponic cultivation in nutrient solution leads to significantly higher soluble sugar and carotenoid contents in stem tips than traditional soil cultivation. Increasing nitrogen supply enhances sweet potato leaf yield, but nitrogen level does not significantly contribute to genotype-by-environment interactions [41,43,44,45].

Water stress profoundly impacts nutritional quality. Through transcriptomic analysis conducted under drought stress during the seedling and root-bulking stages, the drought-tolerant cultivar ‘Wansu 63’ exhibited 7119 to 8811 differentially expressed genes (DEGs) in leaves and roots, respectively. Key genes in the ABA signaling pathway (such as *NCED1*), heat shock proteins, LEA proteins, and sporamin storage proteins were significantly upregulated, alongside the activation of antioxidant defense systems to scavenge reactive oxygen species (ROS). However, drought stress also caused a precipitous decline in biomass to one-quarter of its baseline level, indicating that drought tolerance comes at the expense of vegetative growth and yield [46]. These molecular-level alterations inevitably manifest in the concentrations of soluble sugars, proteins, and antioxidants in the stems and leaves. This suggests that water availability must be considered a critical environmental variable when evaluating the nutritional quality of sweet potato leaves across different regions or climatic years. Furthermore, a study conducted in Tanzania recommended that future research should further investigate the effects of seasonal climates and other environmental conditions on both nutritional and anti-nutritional factors in sweet potato leaves [22]. Altogether, optimized agronomic management can, to a certain extent, directionally modulate the nutritional quality of sweet potato stems and leaves.

#### 2.2.4. Heavy Metal Safety Risks

In addition to cultivar, harvest stage, and cultivation conditions, heavy metal contamination constitutes another critical dimension affecting the safety quality of SPL, with the accumulation of cadmium, lead, arsenic, and chromium directly determining whether SPL can safely enter the human food chain. Cadmium is one of the most extensively studied heavy metals in this context. Its distribution in sweet potato tissues follows a gradient of stem > leaf > tuberous root, and 50–70% of the cadmium in phloem exudate exists as small-molecule chelates, with amino acids and carbohydrates participating in its phloem transport. This phloem-mediated redistribution mechanism implies that even when tuberous roots comply with safety standards, leaves and stems may still exceed regulatory limits due to upward transport via the phloem [47,48,49]. At the mechanistic level, Dong et al. identified the heavy metal-associated isoprenylated plant protein gene *IbHIPP7* in sweet potato. Overexpression of this gene significantly enhanced Cd tolerance in sweet potato and reduced Cd accumulation in the roots and aboveground parts (by 28%, 42%, and 38%, respectively), thereby providing a key genetic target for molecular breeding of low-accumulation cultivars [50].

Cultivar selection exerts a decisive influence on heavy metal accumulation: in some cultivars grown on lightly to moderately cadmium-contaminated soils, leaf and stem cadmium levels may exceed the pollution-free vegetable standard (<0.05 mg·kg^−1^), whereas cultivars such as ‘Shangshu 19’ and ‘Sushu 24’ remain suitable for cultivation even under heavily cadmium- and lead-contaminated conditions. Furthermore, starch-type cultivars exhibit lower cadmium and lead concentrations in tuberous roots than purple-fleshed and table-use cultivars, although the opposite trend is observed in leaves and stems [47,48]. Lead and arsenic concentrations in sweet potato stems are higher than those in leaves and tuberous roots. Field trials on urban soil (lead: 1120 mg·kg^−1^) and orchard soil (lead: 272 mg·kg^−1^; arsenic: 90 mg·kg^−1^) demonstrated that stem consumption should be avoided when sweet potato leaves are grown on soils with elevated lead and arsenic levels [51]. The aforementioned evidence indicates that the heavy metal safety risk associated with SPL is not determined by a single element, but rather results from the combined effects of multi-metal contamination, cultivar differences, soil conditions, and agronomic management practices [47,48,51]. Consequently, the safety quality assessment of SPL must extend beyond the detection of cadmium alone and establish a comprehensive evaluation framework that covers multi-metal contamination, complemented by integrated control measures including cultivar selection, balanced fertilization, and the application of passivators. Only through such an approach can the safe and compliant utilization of SPL as a functional food ingredient be ensured.

## 3. Effects of Processing on Bioactive Compounds and Flavor of SPL

### 3.1. Effects of Processing on Bioactive Components

The processing stability of polyphenols in SPL has been extensively investigated. Liu et al. systematically evaluated the impacts of thermal treatment, ultra-high pressure, pH, light exposure, storage temperature, and simulated digestion on the stability of SPL flavonoids [52]. Their findings indicated that while treatment at 75 °C for 90 min or 600 MPa for 30 min did not significantly alter flavonoid content, exposure to 100 °C for 60 and 90 min reduced antioxidant activity by 20% and 25%, respectively. Under alkaline conditions (pH 7.0–8.0), flavonoid content decreased by approximately 75%, with corresponding antioxidant activity losses of 30% and 47%. Light exposure resulted in a 52% reduction in flavonoid content and a 24% decrease in antioxidant activity. Conversely, no significant changes were observed during storage at −18 °C, 4 °C, or room temperature. Following simulated digestion, the retention rates of flavonoids and antioxidant activity were 45.9% ± 3.6% and 56.2% ± 2.6%, respectively [52]. Using an in vitro digestion model, Chen et al. observed that while SPL polyphenols remain relatively stable in the gastric phase (pH 2), they undergo significant degradation during the transition to the alkaline intestinal environment (pH 7), yielding a bioaccessibility of 13.36% [53]. In a comparative study of five cultivars, Wen et al. found that the “Ecaishu No. 10” variety showed superior phenolic retention after drying, with its polyphenols reaching a maximum bioaccessibility of 48.47% during in vitro digestion [54]. These pronounced inter-cultivar variations suggest that cultivar selection and process optimization are critical for functional food development, depending on the specific processing techniques and target bioactive compounds employed. However, research remains limited concerning the thermal degradation kinetic parameters (such as activation energy) and quantitative pH-sensitivity models for SPL bioactives. Furthermore, the industrial scalability of the aforementioned processing conditions requires further validation.

### 3.2. Processing-Induced Flavor and Palatability Improvement

Despite their nutritional potential, SPLs have not yet gained widespread acceptance as a mainstream leafy vegetable, primarily due to coarse stem fibers, thermal-induced discoloration, and an undesirable flavor profile. Conventional treatments, such as boiling or microwave blanching, remain largely limited to modifying cell wall structure, reducing anti-nutritional factors (e.g., oxalates and phytic acid), and preserving mineral content, while offering limited efficacy in addressing sensory deficiencies [22]. To elucidate the molecular basis of these sensory limitations, integrated metabolomic and transcriptomic analyses have identified 139 metabolites associated with bitterness and astringency in SPL. Flavonoid glycosides and hydroxycinnamic acid derivatives were characterized as the primary bitter constituents, with histidine, alkaloids, and terpenoids also contributing to the bitter profile. Furthermore, 46 structural genes and 24 transcription factors involved in the biosynthesis of these compounds have been identified, providing potential targets for flavor improvement [55].

Based on this chemical understanding, SPLs have been explored as a potential raw material for value-added products, with particular emphasis on tea. Upon drying, SPLs develop characteristic volatile aromas, which render them highly amenable to tea processing. Using green-leaf sweet potato varieties from Tai’an, Shandong Province, response surface methodology was applied to optimize green tea processing parameters—spreading, steaming, stir-frying, and drying—to achieve products with clear infusion color and desirable flavor characteristics [16]. Notably, a compound tea bag formulated with SPL powder, green tea powder, and hawthorn powder at a ratio of 80:15:5, in combination with steam fixation for 3 min, exhibited optimal sensory quality [16]. GC-MS analysis identified 17 volatile compounds in SPL, representing 95.09% of the total volatile fraction, with palmitic acid (47.62%) and linoleic acid (16.62%) as the dominant constituents [16]. Sun-drying and high-temperature baking have been reported to inactivate polyphenol oxidase, inhibit enzymatic browning, eliminate grassy off-odors, and produce roasted aromatic characteristics. Furthermore, processing parameters—including spreading time, steaming duration, stir-frying frequency, and drying temperature—exert significant effects on the sensory attributes of the finished tea product [16].

In addition to conventional processing optimization, emerging molecular strategies offer novel pathways for quality enhancement. Recent research has elucidated a DGP1 protein targeting system that responds to low-phosphorus and red-light signals. Through its TIGR01589 domain, DGP1 binds to key enzymes of glycolysis and the tricarboxylic acid (TCA) cycle and mediates their translocation to the nucleus, thereby redirecting metabolic flux from photorespiration toward the phenylpropanoid pathway. This redirection results in significantly increased accumulation of flavonoids—which contribute to both sensory and health-promoting properties—and soluble sugars, which are positively correlated with sweetness and palatability. This mechanism has been successfully employed to improve the sensory quality of leafy vegetables, including Chinese cabbage [56,57]. The combined implementation of these molecular strategies with advanced processing technologies is anticipated to substantially improve the eating quality of SPLs and thus overcome the barriers to their broader utilization.

## 4. Physiological Activities and Health Functions of SPL

**Figure 2:**
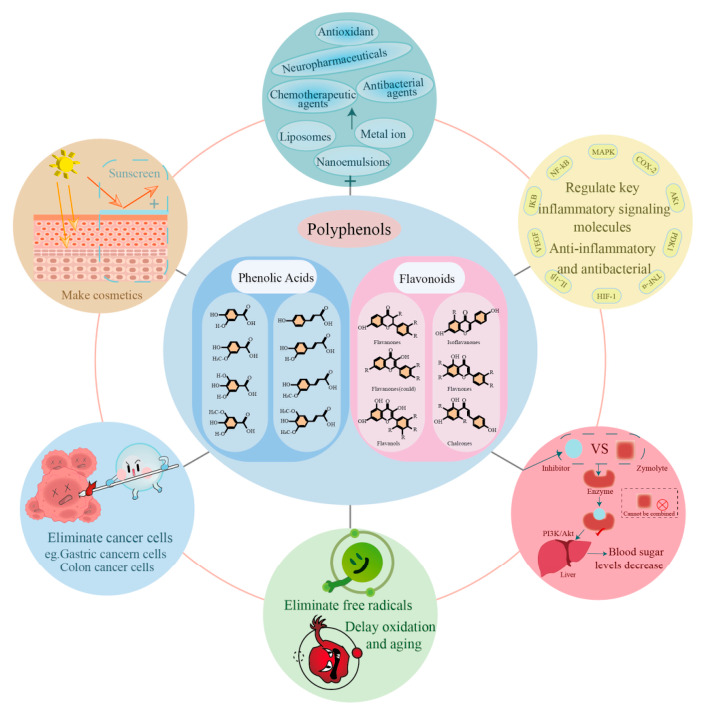
Functional mechanisms of the principal bioactive compounds in sweet potato leaves.

Accumulating evidence indicates that the health benefits of sweet potato leaves are primarily attributable to their core bioactive components, including polyphenols (e.g., chlorogenic acid derivatives), flavonoids, and polysaccharide complexes. The main biological functions of these components are illustrated in Figure 2. (Note: Among them, MAPK: mitogen-activated protein kinases; COX-2: cyclooxygenase 2; Akt: Protein Kinase B; PDK1: phosphoinositide-dependent protein kinase 1; TNF-α: tumor necrosis factor alpha; HIF-1: hypoxia-inducible factor 1; IL-1β: Interleukin-1β; VEGF: vascular endothelial growth factor; IKB: inhibitor of nuclear factor-kappa B alpha; NF-κB: nuclear factor-kappa B).

### 4.1. Antioxidation

Extensive in vitro evaluations of SPL have revealed potent antioxidant capacities across multiple assay platforms: DPPH radical scavenging (EC_50_: 0.19–803.13 μg/mL), FRAP (13.5–705.03 mM TE/g), ORAC (43.72–1174.98 μmol TE/g), and superoxide anion scavenging (EC_50_: 0.10–0.61 mg/mL). Moreover, these overarching antioxidant capacities positively correlate with both total phenolic and total flavonoid contents [12,32,33]. At the molecular level, radical scavenging capacity increases with the degree of caffeoyl acylation. The established hierarchy of antioxidant potency is as follows: 3,4,5-triCQA > diCQA > chlorogenic acid > caffeic acid. Notably, 3,5-dicaffeoylquinic acid (3,5-diCQA)—being the most abundant and exhibiting the strongest monomeric activity—is recognized as the signature antioxidant compound in SPL [12,32,33]. Furthermore, macroscopic variations are pronounced: leaves exhibit significantly greater polyphenol accumulation and antioxidant activity compared to stems and petioles, serving as the primary active sites [33,36]. Cultivar-dependent variations are equally prominent; purple-leaf varieties substantially outperform their green counterparts, primarily driven by the synergistic radical-scavenging effects of anthocyanins. The purification process also plays a critical role, as purified SPL polyphenols demonstrate substantially stronger antioxidant activities than crude extracts [58,59,60,61]. Beyond direct free radical scavenging, studies using H_2_O_2_-induced cellular models have demonstrated that SPL polyphenols indirectly mediate cellular defense by upregulating endogenous antioxidant enzymes (e.g., GSH and SOD) via the Nrf2-ARE signaling pathway [60,62]. Although one human trial reported elevated plasma total antioxidant capacity following the consumption of purple SPL, directly attributing this physiological effect to the absorption of intact polyphenols is scientifically imprecise. Factors such as glycosylation (e.g., rutin) and thermal processing (e.g., steaming) significantly alter phenolic structures, thereby modulating their systemic bioavailability [62,63,64]. Currently, the vast majority of research remains confined to in vitro radical scavenging assays. While these data unequivocally confirm the potent antioxidant properties of SPL polyphenols, in vivo therapeutic efficacy must be interpreted with caution due to their generally low bioavailability, which is heavily influenced by structural modifications and processing methods. Consequently, future investigations must prioritize in vivo metabolism and true systemic absorption.

### 4.2. Hypoglycemic Activity

Both in vitro enzyme inhibition assays and in vivo animal models have confirmed the hypoglycemic efficacy of SPL. Mechanistically, specific polyphenols—such as 3,5-dicaffeoylquinic acid and astragalin—reversibly inhibit α-glucosidase activity by altering its secondary structure, exhibiting IC_50_ values superior to those of the clinical drug acarbose [65,66]. Furthermore, polysaccharide complexes obtained via ultrasound-assisted extraction exhibit potent α-amylase inhibitory effects (IC_50_ = 0.96 mg/mL) [67]. In vivo murine models demonstrate that aqueous SPL extracts activate the PI3K/Akt and AMPK signaling pathways to enhance glucose uptake in peripheral tissues. Additionally, these extracts exert systemic glycemic control by restoring glucose-6-phosphate dehydrogenase (G6PDH) activity and impeding intestinal glucose absorption [29,65,66]. Currently, most animal studies involve acute dosing or short-term interventions, with no data available on long-term toxicity or drug resistance, representing a considerable gap relative to the chronic efficacy assessment required for clinical diabetes management.

### 4.3. Anti-Inflammatory Activity

The anti-inflammatory effects of SPL polyphenols are primarily mediated through the targeted inhibition of the NF-κB signaling pathway. Unlike broad-spectrum non-steroidal anti-inflammatory drugs (NSAIDs), specific phenolic acids in SPL selectively inhibit the phosphorylation of IKK-α and IκB-α. This action blocks the nuclear translocation of the p65 subunit and subsequently downregulates downstream mediators, including iNOS, TNF-α, and IL-1β. Notably, this mechanism does not disrupt the COX-2-PGE_2_ pathway, which theoretically minimizes gastrointestinal side effects [68,69]. Studies utilizing RAW 264.7 macrophages typically employ extract concentrations ranging from 50 to 200 μg/mL [70]. Pharmacokinetic data for dietary polyphenols indicate that systemic plasma concentrations rarely exceed 1–2 μM due to extensive Phase II metabolism (glucuronidation and sulfation) in the liver. Consequently, claims of “systemic anti-inflammatory” benefits derived from high-concentration in vitro studies may be overstated. Instead, the most physiologically plausible target for SPL polyphenols is the gastrointestinal tract itself. As demonstrated in intestinal epithelial cell models (0.2 mg/mL), unabsorbed polyphenols can locally alleviate IL-1β-induced inflammation and restore tight junction proteins, such as ZO-1 and Claudin-1 [71]. This underscores their significant potential in the management of inflammatory bowel disease (IBD) rather than the mitigation of systemic inflammation.

### 4.4. Antitumor Activity

Current evidence regarding the anticancer properties of SPL is strictly confined to in vitro cell lines and preclinical xenograft models. These effects are primarily mediated through the multi-targeted induction of apoptosis and cell cycle arrest. In gastric and colon cancer cells, the pro-apoptotic efficacy of caffeoylquinic acids derived from SPL increases proportionally with the number of caffeoyl groups in their structure. Specifically, these compounds function by downregulating Cyclin D1 and activating Caspase-3 [68]. Furthermore, β-sitosterol-D-glucoside has been demonstrated in mouse models to inhibit the growth of ER-positive breast cancer by upregulating miR-10a and suppressing the PI3K/Akt survival pathway [71]. Given the current lack of clinical validation, the most promising translational value of SPL compounds likely resides in their chemopreventive properties and their synergistic potential as therapeutic adjuvants. Similar to other plant polyphenols, SPL extracts may contribute to mitigating chronic inflammation within the tumor microenvironment. Alternatively, they could serve as chemosensitizers to alleviate the toxic side effects associated with chemotherapeutic agents such as oxaliplatin [72,73].

### 4.5. Other Functions

In addition to polyphenols, SPL-derived pectin functions as a high-viscosity prebiotic fiber that forms a physical barrier within the intestinal tract, thereby delaying starch digestion [74]. However, the in vivo efficacy of this physical barrier is highly dependent on pectin concentration, intestinal peristalsis rates, and chyme rheology—parameters for which quantitative evidence in realistic dietary contexts remains insufficient. Furthermore, because high-viscosity fibers may inadvertently interfere with the absorption of other essential nutrients, the long-term physiological implications of their application require careful evaluation. Unabsorbed polyphenols and dietary fibers also modulate the gut microbiota by promoting the colonization of beneficial strains, such as Lactobacillus brevis SPK2, which exhibits antibacterial activity against pathogens like methicillin-resistant Staphylococcus aureus (MRSA) [75,76]. Nevertheless, these findings are currently limited to in vitro fermentation models. Given the high inter-individual variability of the human gut microbiota and the complex path from microbial modulation to actual anti-infective clinical outcomes, there is currently no direct evidence to suggest that SPL consumption reduces the risk of MRSA infection. Regarding cardiovascular protection, animal studies indicate that absorbed small-molecule phenolic metabolites can chelate transition metal ions and extend the oxidation lag time of low-density lipoprotein (LDL) cholesterol, demonstrating potential anti-atherosclerotic properties [37,63]. However, an extended LDL oxidation lag time is not equivalent to a reduction in clinical endpoints, such as atherosclerotic plaque formation. Current research lacks long-term animal survival data and dynamic monitoring of biomarkers, such as oxidized LDL (oxLDL) antibody titers, to substantiate these cardiovascular benefits.

### 4.6. Metabolism and Bioavailability

The primary bottleneck in translating the in vitro bioactivities of core SPL components (e.g., phenolic acids and anthocyanins) into in vivo health benefits lies in their poor bioaccessibility, rapid metabolism, and low systemic bioavailability. During gastrointestinal digestion, prior to absorption, high-molecular-weight polyphenols are predominantly degraded by the gut microbiota into lower-molecular-weight phenolic metabolites (such as caffeic and ferulic acid derivatives). Consequently, the in vivo physiological effects observed in animal models and human clinical trials are highly likely mediated by these microbial metabolites rather than the intact parent compounds originally present in the plant matrix. To overcome these metabolic limitations, recent research has increasingly focused on novel delivery systems. Strategies such as encapsulating SPL polyphenols within polysaccharide hydrogels (e.g., cellulose nanocrystal-polyacrylamide matrices) or complexing them with plant proteins (e.g., soy protein) have proven effective in protecting these active compounds from premature gastric degradation, thereby facilitating targeted intestinal release [77,78,79]. Such innovations in delivery technologies represent an essential prerequisite for their future translation into high-value clinical applications. The mechanisms of action of these functional substances are systematically summarized in Table 4.

## 5. Application Potential and Safety, Regulatory Status of SPL

### 5.1. Practical Applications of SPL

**Figure 3:**
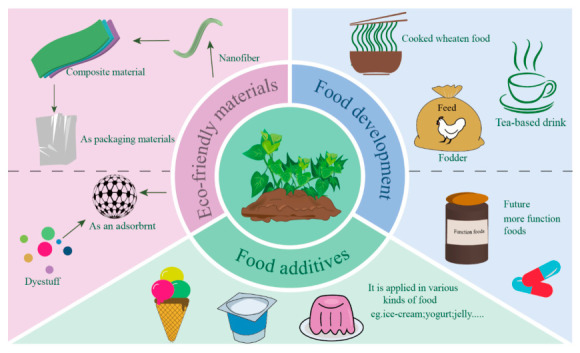
Practical applications potential of SPL.

Based on the upstream attributes and processing outcomes elaborated in the preceding sections, the applications of SPL can be classified into four tiers: food, feed, and industrial materials, ecological agriculture. The following subsections examine each category in turn, with particular emphasis on food applications, which offer the highest value potential yet are subject to the most rigorous safety and quality requirements (Figure 3).

#### 5.1.1. Food Development

Research has demonstrated that the incorporation of 4.5% SPL powder into starch-based noodles significantly improves textural properties and reduces cooking loss. Similarly, additions of 2–10% in steamed bread enhance dietary fiber and mineral content while lowering the estimated glycemic index (eGI) [16,89]. Regarding shelf-life, Pandesal bread containing SPL powder maintains its sensory quality for 7 days under refrigeration (7 °C) [90]. Furthermore, lactic acid-fermented SPL kimchi, subjected to pasteurization (80 °C for 15 min), preserves stable sensory attributes for up to 90 days when stored at 4 °C [91]. Consumer acceptance tests indicate that bread supplemented with 5% SPL powder is most preferred; its overall liking score on a 9-point hedonic scale was significantly higher than that of treatment groups with higher incorporation levels [90]. Nevertheless, high incorporation levels of SPL powder negatively affect product color and flavor due to its crude fiber and inherent flavor compounds, thereby diminishing consumer acceptance. SPL serves as a valuable source of natural food additives. Specifically, caffeoylquinic acid derivatives exhibit potent anti-LDL oxidation effects, offering a natural alternative to synthetic antioxidants (e.g., butylated hydroxytoluene, BHT) for extending the shelf-life of fried foods. These derivatives also function as natural colorants in acidic food systems, such as jellies and yogurts [92]. Additionally, Lactobacillus brevis SPK2, isolated from fermented sweet potato stems, demonstrates superior antioxidant activity (DPPH scavenging rate of 63.57%) and probiotic properties suitable for functional fermented foods [79]. Therefore, beyond direct food additive use, SPL can serve as a source for extracting active compounds, including caffeic acid and flavonoids, to produce functional health foods (e.g., with anti-glycation effects), thus increasing economic value [65].

#### 5.1.2. Feed Application

Research indicates that incorporating 10% SPL powder into broiler diets as a partial replacement for soybean meal has no adverse effects on growth performance or slaughter traits. However, Obakanurhe et al. found that while a 7.5% inclusion level optimizes slaughter traits, levels reaching 15% induce significant histopathological alterations in the heart and liver [93,94]. Furthermore, the addition of 1% hot-air-dried SPL significantly increases body weight, average daily gain (ADG), and meat yellowness, while elevating circulating levels of IGF-1, IL-6, and IFN-γ. This suggests that growth promotion is mediated via enhanced immune responses and the GH/IGF-1 axis [95]. In rabbits, a 20% SPL-cassava leaf meal blend or up to 15% sweet potato peel powder can substitute for corn without compromising growth performance [96,97]. In swine, SPL silage improves ADG under moderate heat stress; the concurrent reduction in heart rate and inflammatory markers indicates enhanced thermoregulation [98]. In emerging aquaculture applications, 5–7.5% SPL extracts mitigate ammonia-induced stress in fish and improve post-transport survival rates in tilapia. However, a comparative study on shrimp revealed that guava leaf powder outperformed SPL, suggesting that SPL is not universally applicable across all aquatic species [99,100]. The nutritional integrity of SPL as a feed ingredient is highly dependent on processing methods: hot-air drying preserves antioxidant properties more effectively than sun-drying, while ensiling successfully reduces anti-nutritional factors while maintaining nutritional value [22,101,102]. Nevertheless, oxalate levels in certain SPL cultivars can reach 3730 mg/100 g, posing risks of renal impairment and calcium deficiency in livestock [22]. Currently, most feeding trials are characterized by short durations (4–8 weeks), leaving a critical gap in long-term safety data. Moreover, consensus guidelines regarding optimal inclusion levels for different species and production systems have yet to be established. Longitudinal feeding trials and systematic dose–response studies are required to address these gaps before SPL can be reliably recommended for commercial feed formulations.

#### 5.1.3. Industrial and Environmental Protection Materials

SPL demonstrates significant potential in industrial and environmental applications, including biodegradable packaging films, dye adsorbents, and biohydrogen production. Cellulose nanocrystals (CNCs) extracted from SPL (average particle size: 191.4 nm; crystallinity: 51.31%) have been incorporated into peanut protein isolate (PPI) to form composite films. The inclusion of 1% CNCs enhanced tensile strength by 40.04% and reduced water vapor permeability by 35% [103]. When applied to strawberry packaging, the spoilage rate was only 30% on day 7, compared to 100% in the control group. This reinforcement mechanism is primarily attributed to hydrogen bonding interactions between the CNCs and the protein matrix. However, the performance of these films under industrial processing conditions, their long-term biodegradability, and their recyclability remain unexplored. In wastewater treatment, pyrolysis-derived biochar from SPL exhibited a high adsorption capacity (750.80 mg/g) for Procion Orange MX-2R dye, along with excellent regeneration performance [104]. Furthermore, KOH-activated carbon derived from SPL has also been investigated for effective dye removal. Regarding biohydrogen production, sweet potato stems proved more suitable for photo-fermentative hydrogen production than leaves. Due to their looser structure, higher cellulose/hemicellulose content (44.6%), and lower crystallinity (29.67%), the stems achieved a cumulative biohydrogen yield of 66.03 mL/g TS—3.59 times higher than that of the leaves—with an energy conversion efficiency increase of 258.93% [105]. While these studies provide innovative pathways for the high-value utilization of SPL, they remain in the preliminary, small-scale validation stage. Future research must prioritize overcoming core bottlenecks such as process scaling, techno-economic analysis, and environmental risk assessment to facilitate industrial-scale implementation.

#### 5.1.4. Ecological Agriculture

Due to its rapid growth, sweet potato effectively suppresses the development and reproduction of invasive plants, such as Bidens pilosa, by outcompeting them for light and nutrients [25]. Beyond direct competition, aqueous extracts of sweet potato leaves exhibit significant allelopathic inhibitory effects. Research conducted by the Yunnan Academy of Agricultural Sciences (2016) demonstrated that these extracts significantly inhibited seed germination, seedling root length, and fresh biomass of five major agricultural weeds: Galinsoga parviflora, Ageratum conyzoides, Bidens pilosa, Digitaria sanguinalis, and Echinochloa crus-galli [106]. This inhibitory effect was dose-dependent; at a concentration of 0.1 g/mL, the inhibition rates for the root length and fresh biomass of *D. sanguinalis* reached 92.04% and 73.33%, respectively. In intercropping systems, Li et al. found that intercropping Kadsura coccinea with sweet potato reduced field weed coverage to 1.23%, a decrease of 52.5 percentage points compared to monoculture. The control rates for weed fresh and dry weights reached 89.2% and 93.45%, respectively. Furthermore, this configuration reduced the incidence of leaf spot disease in K. coccinea by 7.77 percentage points [107]. Collectively, these findings indicate that sweet potato possesses substantial potential for weed suppression and agro-ecosystem regulation across multiple scales, including interspecific competition, allelopathy, and intercropping system optimization.

### 5.2. Safety Assessment and Regulatory Status of SPL

While the conventional consumption of SPL is generally considered safe, a systematic evaluation based on critical empirical evidence remains essential. Toxicological studies have demonstrated that purple SPL extracts exhibit no observable adverse effects in rats during acute (≤2000 mg/kg/day for 14 days) or sub-acute (50–1000 mg/kg/day for 28 days plus a 14-day recovery period) oral administration. Key indicators, including body weight, hematological parameters, and organ histology, remained within normal ranges compared to control groups, supporting the fundamental safety of SPL at standard intake levels [108]. However, some animal studies suggest that prolonged high-dose exposure (>1000 mg/kg/day) may induce inhibitory effects on the thyroid-gonadal axis and potential toxicity to the male reproductive system [109,110]. These findings underscore that safety assessments should not rely solely on acute data but must prioritize chronic toxicity monitoring for long-term exposure. Regarding anti-nutritional factors (ANFs), SPL contains oxalates (308 mg/100 g), phytates (1.44 mg/100 g), cyanide (30.24 mg/100 g), and tannins (0.21 mg/100 g) [111]. Notably, high oxalate intake is associated with an increased risk of urolithiasis (kidney stones); thus, individuals with a history of this condition should avoid excessive consumption. Furthermore, oxalates may interfere with calcium bioavailability, and the consumption of raw or inadequately cooked leaves has been linked to constipation [112]. In terms of its allergenic potential, sweet potato (including its leaves) has been recognized as a food allergen. Velloso et al. utilized immunoblotting to identify specific allergenic protein bands at 54–64 kDa, 19–24 kDa, and 74 kDa, which are capable of triggering IgE-mediated immediate hypersensitivity reactions [113]. Furthermore, case reports have implicated sweet potato as a causative agent for food protein-induced enterocolitis syndrome—a delayed-onset, non-IgE-mediated food allergy primarily affecting infants [114]. Although such cases are relatively infrequent, these findings suggest that individuals with a history of atopy or allergic predispositions should approach the initial consumption of SPL with caution, starting with small quantities to mitigate the risk of potential adverse reactions.

From a regulatory standpoint, the oversight of SPL remains unharmonized globally. While categorized as a conventional food ingredient when consumed as a traditional vegetable, its application as a functional food component or dietary supplement necessitates compliance with specific regulatory frameworks. In the United States, for instance, marketing SPL powder with specific health claims may require a “Generally Recognized as Safe” determination, which must be supported by publicly available scientific evidence and expert consensus [115]. In the Chinese market, its introduction as a “new food raw material” (novel food) must follow the registration protocols established by the National Health Commission (NHC), requiring the submission of comprehensive toxicological safety data and detailed manufacturing specifications [116]. Currently, the toxicological evaluation framework for SPL remains underdeveloped, characterized by a lack of standardized testing protocols. This deficiency hinders the comparability of results across different studies and poses a significant barrier to industrial-scale promotion and commercialization.

## 6. Conclusions and Prospect

This review is the first to systematically integrate the full-chain knowledge of sweet potato leaves—from production to application—and incorporates regulatory frameworks and safety assessments into the analytical system. However, current research still suffers from insufficient data standardization, limited bioavailability evidence, and inadequate safety evaluations. Future efforts should establish standardized reference values, employ multi-omics approaches to improve flavor profiles, develop encapsulation technologies to enhance stability, and validate health benefits through animal experiments and clinical studies. These efforts will ultimately promote the high-value, safe, and sustainable utilization of SPL.

## Figures and Tables

**Table 1:** Standardized nutritional composition of SPL compared with spinach and kale.

Nutritional	SPL Content (Per 100 g FW)	Analytical Method	Spinach (100 g FW)	Kale (100 g FW)	Key Determinants	References
Crude Protein (g)	2.61 ± 0.58–4.85 ± 0.02	Kjeldahl (N × 6.25)	2.86	2.92	Genotype; N-fertilization; Leaf maturity.	[14,18,22,23,25]
Dietary fiber (g)	5.37 ± 0.32–10.03 ± 0.12	AOAC 991.43	2.10	4.10	Leaf maturity.	[14,18,22,23,25,26]
*β*-carotene (mg)	11.18 ± 1.28–69.40 ± 0.48	HPLC	5.60	5.90	Genotype; Light exposure.	[14,18,22,23,25,27]
Vitamin C (mg)	15.84 ± 0.96–81.76 ± 2.08	RP-HPLC; Spectrophotometry; AOAC 967.21	28.10	93.40	Storage duration; Thermal processing.	[18,28,29]
Ca (mg)	80.00 ± 5.92–174.00 ± 2.08	ICP-AES; Atomic absorption spectrometry	99.00	254.00	Soil profile; Genotype; Leaf maturity.	[6,14,18,23,25]
Fe (mg)	1.41 ± 0.53–3.40 ± 1.09	ICP-AES; Atomic absorption spectrometry	2.71	1.60	Genotype; Soil profile; Postharvest handling.	[6,14,18,23,25]
K (mg)	495.00 ± 16.96–955.00 ± 3.36	ICP-AES; Atomic absorption spectrometry	558.00	491.00	Phenological stage; K-Fertilization; Genotype.	[6,14,18,23,25]
Total Oxalate (mg)	463.50–596.90	HPLC-UV	970.00	20.00	Thermal processing; Extraction method.	[22,23,25]

Note: SPL nutrient values were primarily determined on a dry weight basis and uniformly converted to FW based on a water content of 84% from the China Food Composition Table. Nutrient values for raw spinach and raw kale were obtained from the USDA FoodData Central database.

**Table 2:** Summary of polyphenolic components and their contents in SPL.

Component	Content (mg/g DW)	Extraction Method	Reference
Total phenolics	23.30–43.80 mg CAE/g	Different solvents (50%/70%/90% methanol, ethanol, acetone)	[12]
	14.20–171.00.mg ChE/g	80% ethanol	[31]
	18.17–22.80 mg GAE/g	80% ethanol (with 1% HCl)	[32]
	2.90–375.44 mg GAE/g	70% ethanol with ultrasound-assisted extraction	[33]
	10.89–47.52 mg GAE/g	Direct extraction in 85 °C water bath, acid hydrolysis, alkaline hydrolysis	[35]
	28.10–130.00 mg GAE/g	Water extraction	[36]
	26.80 mg CAE/g	70% ethanol with ultrasound-assisted extraction	[37]
	4.20–13.20 mg GAE/g	80% methanol	[38]
Total flavonoids	0.60–3.40 mg QE/g	80% ethanol	[12]
	42.45–67.66 mg QUE/g	80% ethanol (with 1% HCl)	[32]
	1.52–127.12 mg RE/g	70% ethanol with ultrasound-assisted extraction	[33]
	18.00–72.70 mg QE/g	Water extraction	[36]
	0.50–6.60 mg QE/g	80% methanol	[38]
	7.80–23.82 mg QUE/g	80% ethanol (with 1% HCl)	[32]
Anthocyanins	0.11–275.60 mg/g	99% methanol + 1% HCl	[12,32]

**Table 3:** Individual phenolic compounds and their content ranges in SPL.

Class	Component	Content (DW)	Reference
Phenolic acid	CA	0.07–3.76 mg/g	[12,31,35]
	3-CQA	0.03–4.11 mg/g	[12,31]
	3,4-diCQA	0.36–83.28 mg/g	[12,31,32,35]
	3,5-diCQA	0.05–263.64 mg/g	[12,31,32,35]
	4,5-diCQA	0.19–16.51 mg/g	[12,31,35]
	3,4,5-triCQA	0.09–21.62 mg/g	[31,33,35]
	ChE	1.55–9.85 mg/g	[31,35]
Flavonol	Quercetin	0.32–1.73 mg /g	[32,35]
	Morin	3.27–9.38 mg/g	[32]
	Kaempferol	0.06–0.65 mg/g	[35]
Anthocyanidin	Cyanidin	1.44 mg/g	[32]
	Malvidin	0.03 mg/g	[32]
Flavone glycoside	Luteolin-7-O-glucoside	0.75–21.77 mg/g	[33]
Flavonol glycoside	Kaempferol-3-O-glucoside	0.64–18.46 mg/g	[33]
	Hyperoside	2.46–33.58 mg/g	[33]

**Table 4:** Principal bioactive compounds, proposed mechanisms of action, and levels of evidence in SPL.

Bioactivity	Core Bioactive Compounds	Proposed Mechanisms of Action	Research Levels	Reference
Antioxidant	Phenolic acids (e.g., CQA derivatives), Flavonoids	1. Direct radical scavenging via SET and HAT mechanisms. 2. Upregulation of antioxidant enzymes (GSH, SOD) via Nrf2-ARE pathway activation. 3. Attenuation of oxidative cellular damage.	Predominantly In vitro; limited In vivo	[16,36,59,63,80]
Antidiabetic	Polysaccharide complexes, Phenolic acids (e.g., 3,5-diCQA), Flavonoids	1. Competitive inhibition of digestive enzymes (α-amylase, α-glucosidase). 2. Amelioration of insulin resistance via PI3K/Akt and AMPK activation. 3. Stimulation of GLP-1 secretion and G6PDH activity restoration.	In vitro and In vivo (murine models)	[65,66,81]
Anti-inflammatory	Phenolic acids, Flavonoids	1. Downregulation of pro-inflammatory mediators (iNOS, NO). 2. NF-κB pathway suppression (inhibiting IκBα degradation and p65 nuclear translocation) to reduce TNF-α and IL-6 expression.	Primarily In vitro (macrophage/epithelial models)	[68,70,71,82,83]
Chemopreventive	CQA derivatives, Anthocyanins, Luteoloside, Phytosterols	1. Apoptosis induction via CASP3 activation and multi-kinase (EGFR/SRC/KDR) suppression. 2. Modulation of PI3K/Akt and MAPK survival pathways. 3. Cell cycle arrest via c-Jun upregulation and Cyclin D1 downregulation.	In vitro and preclinical In vivo (xenografts)	[71,72,73,84,85]
Photoprotection	Caffeic acid, CQA derivatives	1. Attenuation of UV-induced ROS and enhancement of epidermal SOD. 2. JNK/p38 MAPK pathway inhibition, downregulating MMP-1 and TNF-α.	In vitro (keratinocyte/fibroblast models)	[86,87]
Bioavailability & Delivery	Polyphenol-polysaccharide/protein matrices	1. Gastric protection of polyphenols via encapsulation. 2. Targeted intestinal release and microbial biotransformation into bioavailable low-molecular-weight metabolites.	In vitro (simulated digestion models)	[77,78,79,88]
Cardioprotective	Small-molecule phenolic metabolites	1. Prolongation of LDL oxidation lag time via radical scavenging. 2. Transition metal chelation (Cu^2+^, Fe^3+^) to mitigate lipid peroxidation.	In vitro and In vivo	[37,63]
Antibacterial	Cynarin, Phenolic extracts	1. Disruption of bacterial cell membranes. 2. Inhibition of biofilm formation (e.g., against MRSA).	In vitro	[76]
Prebiotic/Gut Health	Pectin, Unabsorbed polyphenols	1. Formation of an intestinal viscous barrier to delay starch digestion. 2. Microbiome modulation to promote beneficial, antimicrobial strains (Lactobacillus spp.).	In vitro and In vivo	[74,75]

## Data Availability

No new data were created or analyzed in this study. Data sharing is not applicable to this article. A comprehensive literature search was conducted using the Web of Science, Scopus, and PubMed databases, covering publications from January 2000 to December 2025. The search terms included combinations of the following keywords: “sweet potato leaves”, “Ipomoea batatas leaves”, “nutritional composition”, “bioactive compounds”, “polyphenols”, “flavonoids”, “antioxidant activity”, “functional food”, “comprehensive utilization”, and “processing”. Inclusion criteria were: (i) peer-reviewed articles or reviews in English or Chinese on sweet potato stems/leaves related to nutrition, bioactivity, extraction, processing, or applications; (ii) studies with full-text access. Exclusion criteria included: unrelated topics, lack of original data, duplicates, or insufficient methodological detail. After screening, 116 publications were included. Quality assessment was performed using modified JBI and AMSTAR 2 tools, focusing on study design, sample representativeness, analytical validity, and reporting transparency. Disagreements were resolved by author discussion. Expert opinions were also consulted to validate application field classification (e.g., food, feed, biomaterials) and identify industrial bottlenecks. The methodological outcomes are systematically presented throughout the review.

## Abbreviations

The following abbreviations are used in this manuscript:

DW	Dry Weight
iNOS	inducible nitric oxide synthase
DPPH	1,1-Diphenyl-2-picrylhydrazyl radical
MAPK	Mitogen-activated protein kinases
COX-2	Cyclooxygenase 2
Akt	Protein Kinase B
PDK1	Phosphoinositide-dependent protein kinase 1
TNF-α	Tumor necrosis factor alpha
HIF-1	Hypoxia-inducible factor 1;
IL-1β	Interleukin-1β
VEGF	Vascular endothelial growth factor
IKB	Inhibitor of nuclear factor-kappa B alpha
NF-κB	Nuclear factor-kappa B
SOD	Superoxide dismutase
LDL	Low Density Lipoprotein
ROS	Reactive oxygen species
